# Online Workload Allocation via Fog-Fog-Cloud Cooperation to Reduce IoT Task Service Delay

**DOI:** 10.3390/s19183830

**Published:** 2019-09-04

**Authors:** Lei Li, Mian Guo, Lihong Ma, Huiyun Mao, Quansheng Guan

**Affiliations:** 1 School of Electronic and Information Engineering, South China University of Technology, Guangzhou 510641, China; 2 School of Electronic and Information Engineering, Guangdong University of Petrochemical Technology, Maoming 525000, China; 3 School of Computer Science and Engineering, South China University of Technology, Guangzhou 510641, China

**Keywords:** fog system, internet of things, workload allocation, task service delay, Lyapunov drift-plus-penalty

## Abstract

Fog computing has recently emerged as an extension of cloud computing in providing high-performance computing services for delay-sensitive Internet of Things (IoT) applications. By offloading tasks to a geographically proximal fog computing server instead of a remote cloud, the delay performance can be greatly improved. However, some IoT applications may still experience considerable delays, including queuing and computation delays, when huge amounts of tasks instantaneously feed into a resource-limited fog node. Accordingly, the cooperation among geographically close fog nodes and the cloud center is desired in fog computing with the ever-increasing computational demands from IoT applications. This paper investigates a workload allocation scheme in an IoT–fog–cloud cooperation system for reducing task service delay, aiming at satisfying as many as possible delay-sensitive IoT applications’ quality of service (QoS) requirements. To this end, we first formulate the workload allocation problem in an IoT-edge-cloud cooperation system, which suggests optimal workload allocation among local fog node, neighboring fog node, and the cloud center to minimize task service delay. Then, the stability of the IoT-fog-cloud queueing system is theoretically analyzed with Lyapunov drift plus penalty theory. Based on the analytical results, we propose a delay-aware online workload allocation and scheduling (DAOWA) algorithm to achieve the goal of reducing long-term average task serve delay. Theoretical analysis and simulations have been conducted to demonstrate the efficiency of the proposal in task serve delay reduction and IoT-fog-cloud queueing system stability.

## 1. Introduction

The increasing number of Internet of Things (IoT) applications, such as audio recognition, vehicle-to-roadside communications, and virtual reality, often demand a low end-to-end latency between a sensor and a control center [1,2]. These delay-sensitive applications often have stringent task service delay (TSD) requirements, which presents the total delay from the moment at which the task enters the system to when the process is completed.

The development of delay-sensitive IoT applications has presented increasing challenges for the current cloud computing infrastructure. TSD contains not only the computation delay, but also the queuing delay and network delay. Although cloud computing can provide a low-cost, easily expandable, and on-demand high-performance computation service [3,4,5], it relies on huge volumes of data transmissions from the IoT end devices to the remote cloud center, consuming an extremely large amount of network bandwidth resources, as well as causing considerable network delay. Cloud computing has become the bottleneck for the development of delay-sensitive IoT applications [6,7]. 

Fog computing [8,9], which is a middle-tier between the IoT end devices and cloud center, has emerged as a solution to provide high-resilience service quality [10]. It can be considered as an extension of cloud computing, and processes tasks at the edge devices of the network with the aim of preventing the large network delay. Nevertheless, the computation capability of a fog node is limited due to its geographical location (e.g., a pylon or wireless base station) and limited power supply (e.g., solar energy or wind power in remote areas). When the workload of the fog node is heavy, tasks have to be queued at the fog node, and may experience a long queuing delay. In some serious situations, the queuing delay may even exceed the network delay. 

Owing to the limited computation capability of a single fog node, it will be useful to explore and exploit the cooperation among multiple fog nodes and the cloud center to provide lower TSDs. The cooperation of multiple closely connected fog nodes provides a geographically local computation coalition. Thus, the data transmission and network delay between the local fogs and the remote cloud center is reduced significantly [7]. The local computation coalition also reduces the task queueing at a single fog node. 

Workload allocation among fog nodes and the cloud center is a key technique that affects the TSD in QoS provisioning [11]. It determines where a task is serviced in the fog system, and affects both the queueing delay and network delay. However, the dynamic traffic characteristics, and heterogeneous computation capabilities of fog nodes and the cloud center present many challenges for workload allocation. First, the tasks are generated stochastically and the amount of computation also varies for different tasks and over time [12]. An online algorithm would thus be required to solve a workload allocation. Second, owing to the heterogeneous resources of fog nodes and the cloud center, there exists a tradeoff between the queuing delay and the network delay, which complicates workload allocation for tasks. Offloading more tasks to the cloud will increase the network delay, however will decrease the queueing delay at fogs, and vice versa. 

The main focus of this paper is to study the workload allocation problem for an IoT-fog-cloud system with the aim of reducing the TSD. When a task is generated at an IoT end device, it will be delivered to its upstream local fog node. Then, the task can be processed at the local fog node, offloaded to the neighboring fog node, or offloaded to the cloud center. Our goal is to find an optimal workload allocation scheme to allocate workload among the local fog node, neighboring fog nodes, or the cloud center according to the system states, which is formulated as a delay-aware workload allocation problem with the goal of minimizing the TSD. The problem is tackled by a proposed online workload allocation algorithm using Lyapunov drift-plus-penalty theory. 

Our main contributions can be summarized as follows:

- Based on the IoT–fog–cloud system architecture, we present a time-varying queuing model that explicitly considers the heterogeneous computational capability and network delay. Then, a delay-based workload allocation problem is formulated, which suggests the optimal workload allocations among local fog node, neighboring fog nodes, and the cloud center to minimize TSD for tasks.
- We apply the Lyapunov optimization method [13,14] to find out a solution of the workload allocation scheme. Specifically, the drift-plus-penalty properties of the TSD minimization with respect to system stabilization are analyzed. Then, a delay-aware online workload allocation and scheduling algorithm, which enables the local fog node to cooperate with neighboring fog nodes and the cloud center, is proposed. The algorithm can optimize the workload allocation to reduce the average TSD according to the system status online.
- Theoretical analysis and simulation evaluations both illustrate that our proposed algorithm achieves a lower TSD compared with other algorithms.

The remainder of the paper is organized as follows. Section 2 discusses related work. Section 3 introduces the system structure and traffic model. Section 4 describes the problem formulation. Details of the proposed online algorithm are presented in Section 5, in which we also provide a performance analysis of the proposed algorithm. Section 6 presents the simulation evaluation and the results. Finally, Section 7 summarizes the paper.

## 2. Related Work

Fog computing, which provides a flexible computing paradigm with low delay, high security and high energy efficiency, has received an increasing amount of attention in recent years. One of the popular research fields in fog computing is the development of an offloading policy to determine when/where the task can be offloaded and processed by a suitable device in the fog system (e.g., a fog node or the cloud center). 

Xu et al. [15] proposed an online learning algorithm to determine workload offloading in mobile edge computing to minimize the cost of the edge device. Bagula et al. [16] proposed a model for micro-level cost estimation. Based on the model, they proposed a resource allocation algorithm that benefits both the customers and the providers. Similarly, Amoretti et al. [17] proposed a mobile cloud computing simulation model based on queuing network architecture and designed a task offloading policy that optimizes the energy efficiency. Fan and Ansari [18] proposed a workload allocation policy for base stations that considered both the network delay and computing delay to reduce the resource cost and response delay based on an M/M/1 queuing model. Lyu et al. [19] proposed a task offloading policy based on the task delay requirements. Chang et al. [20] also proposed a distributed algorithm based on the alternating direction method of multipliers (ADMM) to determine whether the task should be offloaded to a fog node with the energy efficiency of the user side. Guo et al. [21] provided an energy-efficient dynamic offloading and resource scheduling policy to reduce energy consumption and shorten the application completion time of smart devices in mobile cloud computing. Rahbari and Nickray [22] presented a module placement method based on a classification and regression tree algorithm to offload the task such that the power consumption was minimized. 

Additionally, researchers previously focused on the task offloading based on a three-tier fog system model. Similar to the effort of Li et al. [23], Wu et al. [24] also proposed a three-level mathematical model that included the end devices, middleware consisting of fog nodes, and cloud center. Based on the model, a task offloading algorithm was proposed based on the predicted energy consumption. More importantly, this study considered the computation capability of fog node middleware to be larger than that of the end devices, but smaller than that of the cloud center. Ma et al. [25] proposed an IoT-based fog computing model. Based on the model, a genetic algorithm was proposed for reducing the failure node and energy consumption. Yousefpour et al. [26] proposed a mathematical model of a three-tier fog system to evaluate its performance. They used a threshold method as the task offloading decision to reduce the task delay. Nan et al. [27] also used a queuing model to analyze the performance of the Cloud of Things (CoTs) system consisting of end devices, fog nodes, and a cloud center. They proposed a task offloading policy based on the Lyapunov optimization to minimize the energy cost. Deng et al. [28] also formulated a workload allocation solution that suggested optimal workload allocations between the fog and the cloud and minimizes the power consumption with constrained service delay. Nawrocki and Reszelewski [29] presented two types of offloading task resource usage in a mobile cloud system. Their experimental results showed that resource utilization using the multiple user-one virtual machine (VM) mode was higher than that using the one user-one VM mode, but the performance of the former was lower than that of the latter. Compared with the three-tier fog system, the resource usage of the fog node tier was similar to that of the multiple user-one VM mode because of the limited computation resource, but the cloud center could use the one user-one VM mode to improve the task delay.

This study differs from existing work in the following respects. In this study, workload allocation in a, three-tier fog system (i.e., IoT-fog-cloud fog system) is studied with dynamic workloads, where the computation capability and complicated network delay in different tiers of the system are considered. Thus, we need to find out an online workload allocation scheme among the local fog node, neighboring fog nodes and the cloud center to minimize the task service delay. Then, we propose a fine granular low-complexity workload allocation scheme, which can adaptively switch among the local fog node, neighboring fog nodes, and the cloud center for workload allocation according the system status online. To the best of our knowledge, this is the first effort designed to attain optimal workload allocation for minimal per-task granular service delay in such a three-tier fog system.

## 3. System Description

### 3.1. Internet of Things (IoT)-Fog-Cloud System Model

**Figure 1:**
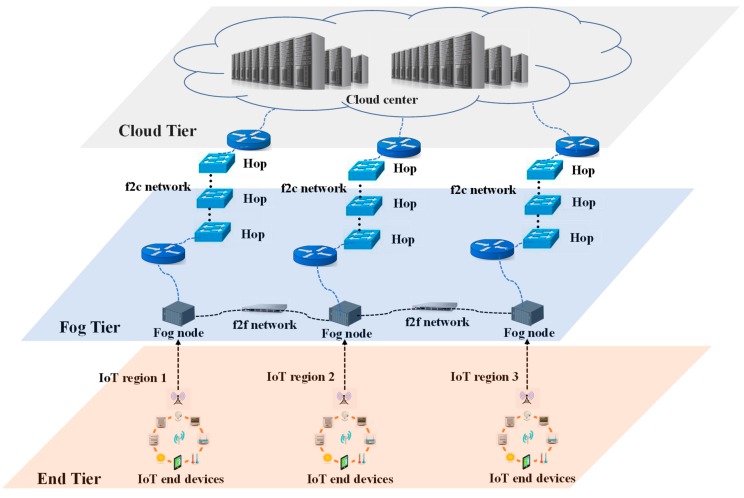
Internet of things (IoT)-fog-cloud system architecture.

As shown in Figure 1, this paper considers an IoT–fog–cloud system, which is divided into three tiers [23] as shown in Figure 1. The end tier consists of multiple IoT devices distributed in several geographically adjacent regions (called IoT regions). The fog nodes form a fog network (f2f network) in the fog tier, where each fog node mainly provides computing services to one downstream IoT region. Thus, a fog node is called the local fog node of the corresponding downstream IoT region [27]. The cloud tier includes the cloud center. A computation task generated in an IoT device can either be computed locally in its upstream fog node, or be offloaded to a neighboring fog through the f2f network, or be offloaded to the cloud center through the fog-to-cloud (f2c) network. 

**Figure 2:**
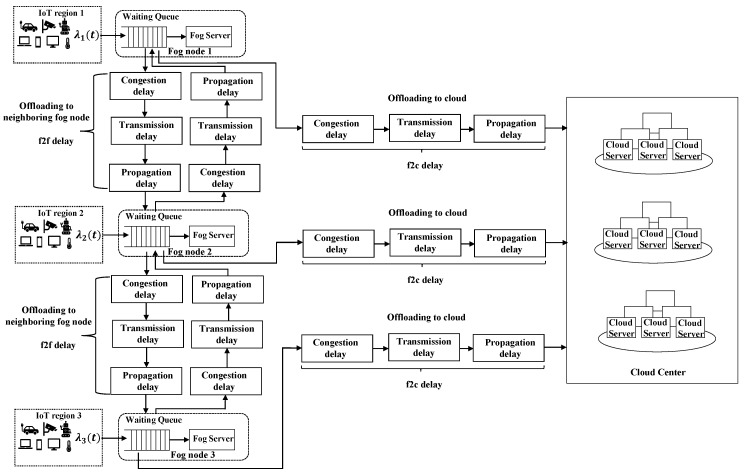
Flow chart showing the processes in the IoT-fog-cloud system.

Note that, when a task is offloaded to a neighboring fog node or the cloud center, it results in three types of network delays, including propagation, transmission, and congestion delays. The propagation delay, which is caused by multi-hop transmissions among routers and switches, can be obtained by using the PING command. The longer the distance is, the longer propagation delay is. The transmission delay is caused by the limited availability of network bandwidth when data is transmitted via the network. When the amount of data to be transmitted increases, the data cache in the network device also increases. Thus, if the amount of data to be transmitted exceeds the bandwidth resource, the remaining data in the network device will introduce the congestion delay in the next time slot. The processing flowchart of the fog system based on the description above is shown in Figure 2.

Assume there are R IoT regions and R fog nodes. Let ℛ={1, …, R}. Assume that fog node j’s downstream IoT region is IoT region j, for ∀j∈ℛ. Let $F_{j}^{fog}$ (j∈ℛ) be the CPU cycle frequency of fog node j. Then, the computation speed $P_{j}$, measuring in million instructions per second (MIPS) [30], is derived by $P_{j}=\frac{F_{j}^{fog}}{10^{6}\;\times \;CPI}$, (where CPI means clock cycle per instruction). The computation resource of the cloud center is assumed to be unlimited in comparison with a fog node, such that any task can be processed immediately after its arrival. Any task is allocated a computation speed $P_{c}=\frac{F^{cloud}}{10^{6}\times CPI}$ immediately after its arrival, where $F^{cloud}$ is the CPU cycle frequency of the cloud center allocated to the task. Assume that $P_{j}<P_{c}$.

### 3.2. Traffic Model

A dynamic discrete-time IoT-fog-cloud system is considered [31]. The arrival process of workloads is as follows: (1) at every time slot, tasks are generated from each IoT region stochastically and independently; (2) in each IoT region, the generated number of tasks per time slot follows an independent and identical distribution (i.i.d); (3) the task length (in million instructions (MI)) and data size (in bits) of each task follow the i.i.d, respectively.

Let $S_{\left(i,j\right)}^{\left(t\right)}$ be the $i^{th}$ task that is generated from IoT region j in time slot t. The task $S_{\left(i,j\right)}^{\left(t\right)}$ is modeled as $\left\{l_{\left(i,j\right)}^{\left(t\right)},\;d_{\left(i,j\right)}^{\left(t\right)}\right\}$, where $l_{\left(i,j\right)}^{\left(t\right)}$ and $d_{\left(i,j\right)}^{\left(t\right)}$ present the task length and data size, respectively.

Let $\chi_{j}\left(t\right)$ be the task space containing the tasks generated from IoT region j in time slot t. Let $\lambda_{j}\left(t\right)$ denote the number of tasks in task space $\chi_{j}\left(t\right)$. Then, $Xw_{j}\left(t\right)=\underset{i\in \chi_{j}\left(t\right)}{\sum }l_{\left(i,j\right)}^{\left(t\right)}$ and $Ys_{j}\left(t\right)=\underset{i\in \chi_{j}\left(t\right)}{\sum }d_{\left(i,j\right)}^{\left(t\right)}$ represent the accumulative computation workloads and data sizes from IoT region j in time slot t, respectively. Let $\bar{\lambda_{j}\left(t\right)}=E\left[\lambda_{j}\left(t\right)\right]$ be the average task generation rate in IoT region j in time slot t, and the long-term average task generation rate is $\bar{\lambda_{j}}=\underset{T\to \infty }{\lim}\frac{1}{T}\sum_{t=0}^{T-1}\bar{\lambda_{j}\left(t\right)}$. Let $\bar{l_{j}\left(t\right)}=E\left[\sum_{i=1}^{\lambda_{j}\left(t\right)\;}l_{\left(i,j\right)}^{\left(t\right)}/\lambda_{j}\left(t\right)\right]$ and $\bar{d_{j}\left(t\right)}=E\left[Ys_{j}\left(t\right)/\lambda_{j}\left(t\right)\right]$ be the expected task length and expected data size in IoT region j in time slot t, respectively. The corresponding long-term expected task instruction length and expected data size of tasks generated in IoT region j are $\bar{l_{j}}=\underset{T\to \infty }{\lim}\frac{1}{T}\sum_{t=0}^{T-1}\bar{l_{j}\left(t\right)}$ and $\bar{d_{j}}=\underset{T\to \infty }{\lim}\frac{1}{T}\sum_{t=0}^{T-1}\bar{d_{j}\left(t\right)}$, respectively.

Let $\chi_{j}^{j}\left(t\right)$, $\chi_{j}^{\left(k\right)}\left(t\right)$ and $\chi_{j}^{c}\left(t\right)$ be the task space containing the tasks that are determined to be processed at the fog node j in time slot t (i.e., local fog node, j∈R), offloaded to neighboring fog node k, and offloaded to the cloud center in time slot t, respectively. $N_{j}^{j}\left(t\right)$, $N_{j}^{\left(k\right)}\left(t\right)$, and $N_{j}^{c}\left(t\right)$ are the corresponding number of tasks in task spaces $\chi_{j}^{j}\left(t\right)$, $\chi_{j}^{\left(k\right)}\left(t\right)$, and $\chi_{j}^{c}\left(t\right)$, respectively. Further, $Xw_{j}^{j}\left(t\right)=\underset{i\in \chi_{j}^{j}\left(t\right)}{\sum }l_{\left(i,j\right)}^{\left(t\right)}$, $Xw_{j}^{\left(k\right)}\left(t\right)=\underset{i\in \chi_{j}^{\left(k\right)}\left(t\right)}{\sum }l_{\left(i,j\right)}^{\left(t\right)}$, and $Xw_{j}^{c}\left(t\right)=\underset{i\in \chi_{j}^{c}\left(t\right)}{\sum }l_{\left(i,j\right)}^{\left(t\right)}$ are the corresponding workloads, respectively. We use K to represent the total space of the neighboring fog nodes of fog node j. Thus, $\chi_{j}^{K}\left(t\right)=\underset{k\in K}{\cup }\chi_{j}^{\left(k\right)}\left(t\right)$ is the total task space containing the tasks that are determined to be offloaded to the neighboring fog nodes of fog node j in time slot t. The corresponding number of tasks in task space $\chi_{j}^{K}\left(t\right)$ is $N_{j}^{K}\left(t\right)=\underset{k\in K}{\sum }N_{j}^{\left(k\right)}\left(t\right)$. The total corresponding workloads that are determined to be allocated to the neighboring fog nodes of fog node j are $Xw_{j}^{K}\left(t\right)=\underset{k\in K}{\sum }Xw_{j}^{\left(k\right)}\left(t\right)$. Additionally, $\chi_{j}\left(t\right)=\chi_{j}^{j}\left(t\right)\cup \chi_{j}^{K}\left(t\right)\cup \chi_{j}^{c}\left(t\right)$.

Since the tasks from IoT region j will finally be processed in the local fog node, or the neighboring fog nodes, or the cloud center, we have:

(1) $$\{\begin{array}{c} \lambda_{j}\left(t\right)=N_{j}^{j}\left(t\right)+N_{j}^{K}\left(t\right)+N_{j}^{c}\left(t\right),\;\\ Xw_{j}\left(t\right)=Xw_{j}^{j}\left(t\right)+Xw_{j}^{K}\left(t\right)+Xw_{j}^{c}\left(t\right). \end{array}$$

### 3.3. Delay Model

#### 3.3.1. Task Service Delay (TSD)

As shown in Figure 2, when the task $S_{\left(i,j\right)}^{\left(t\right)}$ is generated in IoT region j and delivered to the local fog node in time slot t, its TSD is determined by the workload allocation decision. Besides the computation delay, if the task $S_{\left(i,j\right)}^{\left(t\right)}$ is offloaded to the neighboring fog node or the cloud center, the task transmission through the network will cause the network delay. Let $Ct{\left(t\right)}_{\left(i,j\right)}^{\left(m\right)}$ (m∈{j}∪K∪{c}), $Tf_{\left(i,j\right)}^{\left(k\right)}\left(t\right)$ and $Tc_{\left(i,j\right)}\left(t\right)$ be the computation delay, f2f network delay, and f2c network delay, respectively. Thus, the TSD of the task $S_{\left(i,j\right)}^{\left(t\right)}$ can be obtained as:

(2) $$T_{\left(i,j\right)}\left(t\right)=I_{\left(i,j\right)}^{j}\left(t\right)\left(Ct{\left(t\right)}_{\left(i,j\right)}^{\left(j\right)}\right)+\underset{k\in K}{\sum }\left[I_{\left(i,j\right)}^{\left(k\right)}\left(t\right)\cdot \left(Tf_{\left(i,j\right)}^{\left(k\right)}\left(t\right)+Ct{\left(t\right)}_{\left(i,j\right)}^{\left(k\right)}\right)\right]\;+I_{\left(i,j\right)}^{c}\left(t\right)\cdot \left(Tc_{\left(i,j\right)}\left(t\right)+Ct{\left(t\right)}_{\left(i,j\right)}^{\left(c\right)}\right),$$

where $I_{\left(i,j\right)}^{j}\left(t\right)+\underset{k\in K}{\sum }I_{\left(i,j\right)}^{\left(k\right)}\left(t\right)+I_{\left(i,j\right)}^{c}\left(t\right)=1$. When the task $S_{\left(i,j\right)}^{\left(t\right)}$ is determined to be processed at the local fog node (i.e., fog node j), $I_{\left(i,j\right)}^{j}\left(t\right)=1$; otherwise, $I_{\left(i,j\right)}^{j}\left(t\right)=0$; when the task $S_{\left(i,j\right)}^{\left(t\right)}$ is determined to be offloaded to neighboring fog node k, $I_{\left(i,j\right)}^{\left(k\right)}\left(t\right)=1$; otherwise, $I_{\left(i,j\right)}^{\left(k\right)}\left(t\right)=0$; when the task $S_{\left(i,j\right)}^{\left(t\right)}$ is determined to be offloaded to the cloud center, $I_{\left(i,j\right)}^{c}\left(t\right)=1$; otherwise, $I_{\left(i,j\right)}^{c}\left(t\right)=0$. The details of the computation delay and network delay are described as follows.

#### 3.3.2. Computation Delay

Since the computation capability of a fog node is far less than that in the cloud, queueing may happen in a fog node when the workload is heavy. Therefore, we model the computation delays for the fog nodes and the cloud center respectively as follows:

(1) Computation delay at a fog node:

Owing to the limited computation capability of the fog node, we assume that each fog node is a queuing subsystem for buffering the tasks. Let $Q_{j}\left(t\right)$ be the number of tasks being queued in the subsystem of fog node j in time slot t. Assume $Q_{k}\left(0\right)=0$, and based on Equation (1), the queue length $Q_{j}$ is evaluated as follows:

(3) $$Q_{j}\left(t+1\right)=\max\left[Q_{j}\left(t\right)+N_{j}^{e}\left(t\right)+N_{j}^{j}\left(t\right)-\mu_{j}\left(t\right),0\right],$$

where $N_{j}^{e}\left(t\right)$ and $N_{j}^{j}\left(t\right)$ are the number of tasks offloaded from neighboring fog nodes and from downstream IoT region respectively that are determined to be processed at fog node j in time slot t. $\mu_{j}\left(t\right)$ is the number of tasks that are finished at fog node j in time slot t.

Let $A_{j}\left(t\right)$ be the total number of tasks that are determined to be processed at fog node j in time slot t, i.e., $A_{j}\left(t\right)=N_{j}^{e}\left(t\right)+N_{j}^{j}\left(t\right)$. Thus, Equation (2) can be rewritten as follows:

(4) $$Q_{j}\left(t+1\right)=\max\left[Q_{j}\left(t\right)+A_{j}\left(t\right)-\mu_{j}\left(t\right),0\right].$$

Let $\Lambda_{j}\left(t\right)$ be the corresponding task space of $A_{j}\left(t\right)$. Thus, $Xw_{j}^{A}\left(t\right)=\underset{\left(i,n\right)\in \Lambda_{j}\left(t\right)}{\sum }l_{\left(i,n\right)}^{\left(t\right)}$ is the corresponding workload determined to be allocated to fog node j in time slot t. Let ϕ be the length of the time slot. Thus, $P_{j}\cdot \phi$ is the number of instructions processed at fog node j in time slot t.

Let $Qw_{k}\left(t\right)$ be the corresponding workload considering the number of tasks as well as the length of the queues at fog node j in time slot t. Based on Equation (4), we have

(5) $$Qw_{j}\left(t+1\right)=\max\left[Qw_{j}\left(t\right)+Xw_{j}^{A}\left(t\right)-P_{j}\cdot \phi ,0\right].$$

Based on Equation (5), if the task $S_{\left(i,m\right)}^{\left(t\right)}$ is allocated to fog node j in time slot t, its queuing delay can be evaluated as follows:

(6) $$Wq_{\left(i,m\right)}^{\left(j\right)}\left(t\right)=\frac{Qw_{j}\left(t\right)+\sum_{\left(l.n\right)\in \Gamma_{\left(i,m\right)}}l_{\left(l,n\right)}^{\left(t\right)}}{S_{j}},$$

where $\Gamma_{\left(i,m\right)}\left(t\right)$ is the task space containing the tasks that enter into fog node j before the task $S_{\left(i,m\right)}^{\left(t\right)}$ in time slot t. Additionally, when the computation resource becomes available to the task $S_{\left(i,m\right)}^{\left(t\right)}$, its execution time can be calculated as follows:

(7) $$Te_{\left(i,m\right)}^{\left(j\right)}=\frac{l_{\left(i,m\right)}^{\left(t\right)}}{P_{j}}.$$

Accordingly, the computation delay of the task $S_{\left(i,m\right)}^{\left(t\right)}$ at fog node j can be evaluated as follows:

(8) $$Ct{\left(t\right)}_{\left(i,m\right)}^{\left(j\right)}=Wq_{\left(i,m\right)}^{\left(j\right)}\left(t\right)+Te_{\left(i,m\right)}^{\left(j\right)}.$$

(2) Computation delay in the cloud center: 

By contrast, since any task can be processed immediately after its arrival, the computation delay of the task $S_{\left(i,m\right)}^{\left(t\right)}$ at the cloud center equals to the execution time, which can be calculated as follows:

(9) $$Ct{\left(t\right)}_{\left(i,m\right)}^{c}=Te_{\left(i,m\right)}^{c}=\frac{l_{\left(i,m\right)}^{\left(t\right)}}{P_{c}}.$$

#### 3.3.3. Network Delay

As shown in Figure 2, there are two types of network transmission paths: the f2f and f2c network paths. We model these two types of network delay as follows, respectively.

(1) f2f delay:

Let $\{Bw_{j}^{\left(k\right)}\;,Tp_{j}^{\left(k\right)}$} denote the parameters of the network that transmits the data from fog node j to fog node k, where $Bw_{j}^{\left(k\right)}$, $Tp_{j}^{\left(k\right)}$ represent the bandwidth and the propagation delay, respectively. 

Since the f2f network is generally a one-hop and bandwidth-constrained network in comparison with the f2c network, network congestion may happen in the f2f network in a traffic-bursting period. We use $G_{j}^{\left(k\right)}\left(t\right)$ to represent the remaining data to be transmitted in this link at the beginning of time slot t. Specially, $G_{j}^{\left(k\right)}\left(t\right)=0$ indicates no congestion. Thus, the congestion delay contributing to the f2f delay is calculated as:

(10) $$Tg_{\left(i,j\right)}^{\left(k\right)}\left(t\right)=\frac{G_{j}^{\left(k\right)}\left(t\right)+\sum_{n\in Z_{\left(j\right)}^{\left(k\right)}\left(t\right)}d_{\left(n,j\right)}^{\left(t\right)}}{Bw_{j}^{\left(k\right)}},\;$$

where $Z_{\left(j\right)}^{\left(k\right)}\left(t\right)$ contains the tasks that will be transmitted from fog node j to fog node k before the task $S_{\left(i,j\right)}^{\left(t\right)}$ in time slot t. 

The transmission delay of the task $S_{\left(i,j\right)}^{\left(t\right)}$ can be calculated as follows:

(11) $$Ts_{\left(i,j\right)}^{\left(k\right)}\left(t\right)=\frac{d_{\left(i,j\right)}^{\left(t\right)}}{Bw_{j}^{\left(k\right)}}.\;$$

Therefore, if the task $S_{\left(i,j\right)}^{\left(t\right)}$ is transmitted from fog node j to fog node k, its f2f delay can be evaluated as follows:

(12) $$Tf_{\left(i,j\right)}^{\left(k\right)}\left(t\right)=Tg_{\left(i,j\right)}^{\left(k\right)}\left(t\right)+Ts_{\left(i,j\right)}^{\left(k\right)}\left(t\right)+Tp_{j}^{\left(k\right)}.\;$$

(2) f2c delay:

Let $\{B_{j}^{c}\;,Tp_{j}^{c}$} represent the bandwidth and propagation delay of the f2c. Since the network resource of the datacenter network is far sufficient in comparison with the edge network (e.g., f2f network), it is reasonable to assume that $Bw_{j}^{k}<Bw_{j}^{c}$ and $Tp_{j}^{\left(k\right)}<Tp_{j}^{c}$ [32]. Similar to the propagation model in Section 3.3.2-(1), we use again $G_{j}^{c}\left(t\right)$ to represent the remaining data to be transmitted in the link from fog node j to the cloud center at the beginning of time slot t. Thus, the congestion delay can be calculated as:

(13) $$Tg_{\left(i,j\right)}^{c}\left(t\right)=\frac{G_{j}^{c}\left(t\right)+\sum_{n\in Z_{\left(j\right)}^{c}\left(t\right)}d_{\left(n,j\right)}^{\left(t\right)}}{Bw_{j}^{c}},\;$$

where $Z_{\left(j\right)}^{c}\left(t\right)$ contains the tasks that will be transmitted from fog node j to the cloud center before the task $S_{\left(i,j\right)}^{\left(t\right)}$. The transmission delay can be calculated as follows:

(14) $$Ts_{\left(i,j\right)}^{c}\left(t\right)=\frac{d_{\left(i,j\right)}^{\left(t\right)}}{Bw_{j}^{c}}.$$

Thus, if the task $S_{\left(i,j\right)}^{\left(t\right)}$ is offloaded from fog node j to the cloud center, its f2c delay can be evaluated as follows:

(15) $$Tc_{\left(i,j\right)}\left(t\right)=Tg_{\left(i,j\right)}^{c}\left(t\right)+Ts_{\left(i,j\right)}^{c}\left(t\right)+Tp_{j}^{c}.\;$$

#### 3.3.4. Average Task Service Delay

In a lossless system, based on Equation (2), the average TSD of tasks generated from all regions in time slot t is derived by:

(16) $$T_{avg}\left(t\right)=\frac{\sum_{j=1}^{R}\sum_{i=1}^{\lambda_{j}\left(t\right)}T_{\left(i,j\right)}\left(t\right)}{\sum_{j=1}^{R}\lambda_{j}\left(t\right)}.\;$$

The long-term average TSD of tasks generated from all regions can be calculated by:

(17) $$\bar{T_{avg}}=\underset{T\to \infty }{\lim}\frac{1}{T}\sum_{t=0}^{T-1}E\left[T_{avg}\left(t\right)\right].\;$$

## 4. Problem Formulation and Transformation

### 4.1. Problem Formulation

Our goal is to reduce the value of $\bar{T_{avg}}$ in Equation (17). At the same time, to avoid an extremely long queuing delay, the queuing system should be stable, where the stability of the fog system is defined as follows.

**Definition** **1.** *(Stability of a fog system). A fog system is stable if queue vector*Q(t)*and workload vector*Qw(t)*are both stable, where*$Q\left(t\right)=\left[Q_{1}\left(t\right),\;Q_{2}\left(t\right),\;\ldots ,\;Q_{R}\left(t\right)\right]$*and*$Qw\left(t\right)=\left[Qw_{1}\left(t\right),\;Qw_{2}\left(t\right),\ldots ,Qw_{R}\left(t\right)\right]$, respectively, i.e.,

(18) $$\{\begin{array}{c} \underset{T\to \infty }{\lim}\frac{1}{T}\sum_{t=0}^{T-1}E\left[\sum_{j=1}^{R}Q_{j}\left(t\right)\right]<\infty \\ \underset{T\to \infty }{\lim}\frac{1}{T}\sum_{t=0}^{T-1}E\left[\sum_{j=1}^{R}Qw_{j}\left(t\right)\right]<\infty \end{array}.$$

Let $\pi_{\left(i,j\right)}\left(t\right)=\left(\begin{array}{ccccc} I_{i}^{j}\left(t\right) & \ldots & I_{i}^{\left(k\right)}\left(t\right) & \ldots & I_{i}^{c}\left(t\right) \end{array}\right)$ be the decision vector for the task $S_{\left(i,j\right)}^{\left(t\right)}$. Then, the workload allocation decisions for $\lambda_{j}\left(t\right)$ arriving tasks at time slot t at fog node j can be represented as follows:

(19) $$\pi^{\left(j\right)}\left(t\right)=\left(\pi_{\left(1,j\right)}\left(t\right),\;\pi_{\left(2,j\right)}\left(t\right),\ldots ,\;\pi_{\left(\lambda_{j}\left(t\right),j\right)}\left(t\right)\right).\;$$

The decision vector for all tasks generated from all fog nodes is expressed as:

(20) $$\pi \left(t\right)=\left(\pi^{\left(1\right)}\left(t\right),\;\ldots ,\pi^{\left(j\right)}\left(t\right),\ldots ,\pi^{\left(R\right)}\left(t\right)\right).\;$$

Then, according to the definitions of $N_{j}^{j}\left(t\right)$, $N_{j}^{\left(k\right)}\left(t\right)$, and $N_{j}^{c}\left(t\right)$ as well as $Xw_{j}^{j}\left(t\right)$, $Xw_{j}^{\left(k\right)}\left(t\right)$ and $Xw_{j}^{c}\left(t\right)$, and $Xw_{j}^{c}\left(t\right)$ in Equation (1), we have

(21) $$\{\begin{array}{c} N_{j}^{j}\left(t\right)=\underset{i\in \chi_{j}\left(t\right)}{\sum }I_{\left(i,j\right)}^{j}\left(t\right),\;\\ N_{j}^{\left(k\right)}\left(t\right)=\underset{i\in \chi_{j}\left(t\right)}{\sum }I_{\left(i,j\right)}^{\left(k\right)}\left(t\right),\\ N_{j}^{c}\left(t\right)=\underset{i\in \chi_{j}\left(t\right)}{\sum }I_{\left(i,j\right)}^{c}\left(t\right). \end{array}$$

and

(22) $$\{\begin{array}{c} Xw_{j}^{j}\left(t\right)=\sum_{i\in \chi_{j}\left(t\right)}I_{\left(i,j\right)}^{j}\left(t\right)\cdot l_{\left(i,j\right)}^{\left(t\right)},\\ Xw_{j}^{\left(k\right)}\left(t\right)=\sum_{i\in \chi_{j}\left(t\right)}I_{\left(i,j\right)}^{\left(k\right)}\left(t\right)\cdot l_{\left(i,j\right)}^{\left(t\right)},\\ Xw_{j}^{c}\left(t\right)=\sum_{i\in \chi_{j}\left(t\right)}I_{\left(i,j\right)}^{c}\left(t\right)\cdot l_{\left(i,j\right)}^{\left(t\right)}. \end{array}$$

Since, $E\left[T_{avg}\left(t\right)\right]$ in Equation (17) is determined by π(t), $E\left[T_{avg}\left(t\right)\right]$ can be represented as $E\left[T_{avg}\left(\pi \left(t\right)\right)\right]$. Therefore, the workload allocation problem for minimizing the TSD in the fog system can be formulated as:

(23) $$\begin{array}{ll} \mathrm{Minimize}\bar{T_{avg}} & =\underset{T\to \infty }{\lim}\frac{1}{T}\sum_{t=0}^{T-1}E\left[T_{avg}\left(\pi \left(t\right)\right)\right]\\ & =\underset{T\to \infty }{\lim}\frac{1}{T}\sum_{t=0}^{T-1}\frac{\sum_{j=1}^{R}\sum_{i=1}^{\lambda_{j}\left(t\right)}T_{\left(i,j\right)}\left(t\right)}{\sum_{j=1}^{R}\lambda_{j}\left(t\right)},\;\forall t\in \left\{0,1,2,\ldots ,\infty \right\},\; \end{array}$$

*s.t.* Equations (1), (18), (21) and (22).

where Equation (23) follows Equation (17); Equation (18) is the stability constraint of the system, Equation (1) is the traffic constraint; Equations (21) and (22) follow the definition of π(t). 

The above problem is equivalent to determining a sequential optimal $\pi^{*}\left(t\right)$ for t=0, 1, …, ∞ to achieve the objective of minimizing E[·], where $\pi^{*}\left(t\right)=\left(\pi^{\left(1\right)*}\left(t\right),\;\ldots ,\pi^{\left(j\right)*}\left(t\right),\ldots ,\pi^{\left(R\right)*}\left(t\right)\right)$. 

### 4.2. Problem Transformation

To achieve the objective in Equation (23), we can transfer the problem by minimizing the average TSD in each time slot. Then, the problem in Equation (23) can be transformed as follows:

(24) $$\mathrm{Minimize}\;E\left[T_{avg}\left(\pi \left(t\right)\right)\right],\;\forall t\in \left\{0,\;1,\;2,\ldots ,\infty \right\},\;$$

*s.t.* Equations (1), (18), (21) and (22).

Based on Definition 1 in Section 4.1, we have the following lemma.

**Lemma** **1.** Q(t)*and*Qw(t)*are both stable if*$\bar{Qw_{j}}<\infty$, *where*$\bar{Qw_{j}}$*is the long-term average length of*$Qw_{j}\left(t\right)$.

**Proof.**  According to $\bar{Qw_{j}}<\infty$, we assume that $\bar{Qw_{j}}<C<\infty$, where C is a finite constant. Then, we have:

$$\underset{T\to \infty }{\lim}\frac{1}{T}\sum_{t=0}^{T-1}E\left[\sum_{j=1}^{R}Qw_{j}\left(t\right)\right]=\sum_{j=1}^{R}E[\underset{T\to \infty }{\lim}\frac{1}{T}\sum_{t=0}^{T-1}Qw_{j}\left(t\right)]<\sum_{j=1}^{R}\bar{Qw_{j}}<\sum_{j=1}^{R}C<\infty .$$

Thus, Qw(t) can remain stable if $\bar{Qw_{j}}<\infty$. Let $\bar{l^{j}}$ denote the long-term average length of an instruction of a task processed by fog node j. If $\bar{Qw_{j}}<\infty$, the length of a task instruction is finite. Further, in the system, there is at least one task’s instruction length exceeds 0; thus, the average length of a task instruction is larger than 0. Thus, we have $0<L<\bar{l^{j}}<\infty$, where L is a finite constant greater than 0. We obtain:

$$\underset{T\to \infty }{\lim}\frac{1}{T}\sum_{t=0}^{T-1}E\left[\sum_{j=1}^{R}Q_{j}\left(t\right)\right]=\sum_{j=1}^{R}\frac{E\left[\underset{T\to \infty }{\lim}\frac{1}{T}\sum_{t=0}^{T-1}Qw_{j}\left(t\right)\right]}{\bar{l^{j}}}\leq \sum_{j=1}^{R}\frac{C}{L}<\infty .$$

Hence, Q(t) and Qw(t) can remain stable if $\bar{Qw_{j}}<\infty$, which proves Lemma 1. □

Using Lemma 1, the problem in Equation (24) can be transformed as:

(25) $$\mathrm{Minimize}\;E\left[T_{avg}\left(\pi \left(t\right)\right)\right]=\frac{\sum_{j=1}^{R}\sum_{i=1}^{\lambda_{j}\left(t\right)}T_{\left(i,j\right)}\left(t\right)}{\sum_{j=1}^{R}\lambda_{j}\left(t\right)},\;\forall t\in \{0,1,2,\ldots ,\infty \},\;$$

$$s.t.\;\bar{Qw_{j}}<\infty ,\forall j\in \left\{1,2,\ldots ,R\right\},\;\mathrm{Equations}\;(1),\;(21)\;\mathrm{and}\;(22).$$

## 5. Delay-Aware Workload Allocation and Task-Scheduling Scheme

### 5.1. Lyapunov Drift-Plus-Penalty

As mentioned above, to avoid extremely long queuing delay at the fog node, the workload allocation policy needs to ensure that the queuing system of the fog nodes remains stable. The Lyapunov optimization [13,14], which is central to the study of the optimal control in queuing networks, has been used extensively in control theory to ensure the stability of different forms of systems. We again use the Lyapunov optimization technique to find an efficient online workload offloading scheme to maintain the stability of the queuing fog system. Based on Equation (5), the Lyapunov function of the fog nodes in the system is expressed as follows:

(26) $$L\left(t\right)=\frac{1}{2}\sum_{j=1}^{R}{\left[Qw_{j}\left(t\right)\right]}^{2}.\;$$

The one-step conditional Lyapunov drift, which represents the difference in the Lyapunov function in two consecutive time slots, is:

(27) ΔL(t)=E[L(t+1)−L(t)|Qw(t)], 

where $Qw\left(t\right)=\left[Qw_{1}\left(t\right),\;Qw_{2}\left(t\right),\ldots ,Qw_{R}\left(t\right)\right]$. We have the following theorem.

**Theorem** **1.** *In every time slot*t*, for any value of*Qw(t)*, and under any policy, the Lyapunov drift of*Qw(t) satisfies:

(28) $$\Delta L\left(t\right)\leq B+E\left[\sum_{j=1}^{R}\left(Qw_{j}\left(t\right)\cdot \left(Xw_{j}^{A}\left(t\right)-P_{j}\cdot \phi \right)\right)|Qw\left(t\right)\right],\;$$

*where* B *is a finite constant*.

The proof is described in Appendix A.

Our goal is to determine a sequential optimal workload allocation decision $\pi^{*}\left(t\right)$ for t=0,1,…,∞ to achieve the objective in Equation (25). Following the drift-plus-penalty technique, we can minimize the upper bound of the following expression in each time slot to optimize and stabilize all queues,

(29) $$\Delta L\left(t\right)+V\cdot E\left[T_{avg}\left(\pi \left(t\right)\right)|Qw\left(t\right)\right],\;$$

where V is a non-negative control parameter that is chosen as desired and affects the queuing workload and the TSD tradeoff.

Accordingly, we add $E\left[T_{avg}\left(\pi \left(t\right)\right)\right]$ as penalty to both sides of the Lyapunov drift in Equation (28) as follows:

(30) $$\Delta L\left(t\right)+V\cdot E\left[T_{avg}\left(\pi \left(t\right)\right)|Qw\left(t\right)\right]\leq \;B+E\left[\sum_{j=1}^{R}\left(Qw_{j}\left(t\right)\cdot \left(Xw_{j}^{A}\left(t\right)-P_{j}\cdot \phi \right)\right)|Qw\left(t\right)\right]+V\cdot E\left[T_{avg}\left(\pi \left(t\right)\right)|Qw\left(t\right)\right].\;$$

### 5.2. Delay-Aware Online Workload Allocation and Task-Scheduling Algorithm

Furthermore, according to the Lyapunov drift theory, if the Lyapunov drift-plus-penalty in Equation (29) is close to zero, or even a negative value, this implies that the queue length would be stabilized, and the TSD would be reduced. Thus, based on Equation (30), the optimization problem can be formulated as minimizing a bound on the following drift-plus-penalty:

(31) $$\mathrm{Minimize}E\left[\sum_{j=1}^{R}\left(Qw_{j}\left(t\right)\cdot \left(Xw_{j}^{A}\left(t\right)-P_{j}\cdot \phi \right)\right)|Qw\left(t\right)\right]+V\cdot E\left[T_{avg}\left(\pi \left(t\right)\right)|Qw\left(t\right)\right].\;$$

Then, Equation (31) can be rewritten as follows:

(32) $$\mathrm{Minimize}E\left[\sum_{j=1}^{R}\left(Qw_{j}\left(t\right)\cdot \left(Xw_{j}^{A}\left(t\right)-P_{j}\cdot \phi \right)\right)+V\cdot T_{avg}\left(\pi \left(t\right)\right)|Qw\left(t\right)\right].\;$$

Note that, although $P_{j}\;\phi$ in Equation (32) can affect $Qw^{j}\left(t\right)$, it is independent from the workload allocated to the fog node. Then, we can transform the problem in Equation (32) as follows:

(33) $$\mathrm{Minimize}\;\sum_{j=1}^{R}\left[Qw_{j}\left(t\right)\cdot Xw_{j}^{A}\left(t\right)\right]+V\cdot T_{avg}\left(\pi \left(t\right)\right).\;$$

Two methods are available to achieve the objective in Equation (33): the central management framework and the distributed management framework. Central management relies on a central node to manage the workload allocation of all fog nodes in the fog system. However, the central node requires information about the system status. Thus, the central node needs to traverse all fog nodes to determine the workload allocation of the tasks, which would cause frequent information broadcasts about the system status. Because of the need for the traversal operation involving all fog nodes, it would be difficult to complete the frequent broadcasts in one time slot. Similarly, the workload allocation command sent from the central node to the other fog nodes cannot be guaranteed in real time. Furthermore, the delay incurred when sending the command and the traversal of all fog nodes also increases the task-waiting delay. This indicates that the central management framework is difficult to be deployed and used in a real situation. 

Thus, we propose a distributed management framework to find out the solution. Specifically, based on Equation (33), we propose the delay-aware online workload allocation (DAOWA) algorithm to determine the sequential optimal workload allocations $\pi^{\left(j\right)*}\left(t\right)$ for fog node j for t=0, 1, …, ∞ to minimize the drift-plus-penalty in every time slot, such that we achieve the goal of stabilizing the queue length and reducing the TSD. The pseudocode of the DAOWA algorithm is detailed in Algorithm 1, which follows:

Each fog node broadcasts its own status information to its neighboring fog nodes immediately after all processing schemes are put into operation.

(1) In time slot *t*, to operate the DAOWA algorithm, each fog node needs to evaluate the information of its neighboring fog nodes based on information that was previously broadcasted. The fog node updates the evaluation only if it receives the newly broadcasted information.
(1) In each time slot, each fog node manages its own workload allocation independently based on the evaluation of its own status information and the neighboring fog node status information.

Furthermore, in a distributed management framework, because each fog node is responsible for its own workload allocation, we use Equation (34) instead of Equation (33) in Algorithm 1. Each fog node can only obtain the information (e.g., Qw(t) or the workload allocation decision) of its neighboring fog nodes at most once in a slot; thus, fog node j uses $Xw_{j}^{j}\left(t\right)$ and $Xw_{j}^{\left(k\right)}\left(t\right)$ in Equations (35) and (36) to approximate the workload of the local fog node and neighboring fog node k in time slot t, respectively. Similarly, fog node j uses the average TSD of tasks generated from the local region in time slot *t*, i.e., $T_{avg}^{\left(j\right)}\left(t\right)$ ($T_{avg}^{\left(j\right)}\left(t\right)=\frac{\sum_{i=1}^{\lambda_{j}\left(t\right)}T_{\left(i,j\right)}\left(t\right)}{\lambda_{j}\left(t\right)}$) in Equation (34), to approximate $T_{avg}\left(\pi \left(t\right)\right)$.

**Algorithm 1** Delay-aware online workload allocation (DAOWA) algorithm**Input:**$\left[Qw_{j}\left(t\right),\;\ldots ,\;Qw_{k}\left(t\right),\ldots \right]\;\mathrm{and}\;\left[Xw_{j}^{A}\left(t\right),\;\ldots ,Xw_{k}^{A}\left(t\right),\ldots \right],\;\forall j\in ℛ,k\in K$, K∈ℛ and j≠k, where ℛ and K are the fog node space and the space of the neighboring fog node of fog node j, respectively.
**1)** **Initialization:**$Xw_{j}^{A}\left(t\right)=0$, …, $Xw_{k}^{A}\left(t\right)=0$,….**2)** **Decision process:****For** task arrival in time slot t, **do**Choose $\pi^{j*}\left(t\right)$ as the optimal decision for fog node j as follows:
(34)$$\mathrm{Minimize}\;\sum_{m\in \left\{j,K\right\}}\left[Qw_{m}\left(t\right)\cdot Xw_{m}^{A}\left(t\right)\right]+V\cdot T_{avg}^{\left(j\right)}\left(t\right)$$    s.t. (1), (21), (22),
(35)$$Xw_{j}^{A}\left(t\right)=Xw_{j}^{j}\left(t\right)=\sum_{\chi_{j}\left(t\right)}I_{\left(i,j\right)}^{j}\left(t\right)\cdot l_{\left(i,j\right)}^{\left(t\right)},$$
(36)$$Xw_{k}^{A}\left(t\right)=Xw_{j}^{\left(k\right)}\left(t\right)=\sum_{i\in \chi_{j}\left(t\right)}I_{\left(i,j\right)}^{\left(k\right)}\left(t\right)\cdot l_{\left(i,j\right)}^{(t).}$$**3)** **Processing the decisions:**Observer $\pi_{\left(i,j\right)}\left(t\right)$ in $\pi^{\left(j\right)*}\left(t\right)$, **do**
**a)** **If**$I_{\left(i,j\right)}^{j}\left(t\right)=1$, **do** Buffer the task $S_{\left(i,j\right)}^{\left(t\right)}$ into the local fog node (i.e., fog node j);**b)** **Else if**$I_{\left(i,j\right)}^{\left(k\right)}\left(t\right)=1$, **do** Transmit the task $S_{\left(i,j\right)}^{\left(t\right)}$ to neighboring fog node k;**c)** **Else if**$I_{\left(i,j\right)}^{c}\left(t\right)=1$, **do** Transmit the task $S_{\left(i,j\right)}^{\left(t\right)}$ to the cloud center.

**Output:**
$\pi^{j*}\left(t\right)$.

In addition to the workload allocation process, the task undergoes a scheduling process in the fog system. The workload allocation process determines where to process the task, whereas the scheduling process services the task based on the workload allocation decision. Therefore, based on Algorithm 1, we proposed an online workload allocation and task-scheduling algorithm, namely DAOWA-based workload allocation and task-scheduling algorithm, which is described in Algorithm 2.

**Algorithm 2** DAOWA-based workload allocation and task scheduling algorithm
**1)** 
**Initialization:**
**For** each fog node, **do** $Qw_{j}\left(0\right)=0$, …,$\;Qw_{k}\left(t\right)=0$, …, ∀j∈ℛ,k∈K, and j≠k, where ℛ and K are the fog node space and the space of the neighboring fog node of fog node j, respectively.**2)** 
**The task workload allocation process:**
**For** each time slot, **do****a)** **For** all fog nodes in parallel:**i)** **Initialization:**Update the queuing status evaluation of the neighboring fog nodes, i.e., $\;Qw_{k}\left(t\right)$,∀k∈K, according to the previous broadcast.**ii)** **Workload allocation process:**Run Algorithm 1 for fog node j to obtain $\pi^{\left(j\right)*}\left(t\right)$.**3)** 
**Task scheduling process:**
**For** each time slot, **do****a)** **For** all fog nodes in parallel:**i)** Schedule the workload of task according to $\pi^{\left(j\right)*}\left(t\right)$ which is obtained from Algorithm 1;**ii)** Process the waiting tasks with $P_{j}\cdot \phi$ in the first-in-first-out (FIFO) discipline;**iii)** **If** the fog node receives tasks through the f2f network transmission, **do** Buffer the tasks in the waiting queue of the node in the $j^{th}$ region. Update $Qw_{j}\left(t\right)$, $Q_{j}\left(t\right)$ and congestion events with Equations (4) and (5).**b)** **For** the cloud center:**If** the cloud center receives tasks via f2c network transmission, **do** Initiate the VMs with the same number of tasks to process the tasks.**4)** 
**Broadcast process:**
 Each fog node broadcasts the information of the $Qw_{j}\left(t\right)$ to its neighboring fog nodes.


### 5.3. Performance Analysis 

This subsection further discusses the performance of the DAOWA algorithm in terms of the average queuing length of a workload and the average TSD. Let $\pi^{*}\left(t\right)=\left(\pi^{\left(1\right)*}\left(t\right),\;\ldots ,\pi^{\left(j\right)*}\left(t\right),\ldots ,\pi^{\left(R\right)*}\left(t\right)\right)$ be the optimal decision based on the DAOWA algorithm, $Xw_{j}^{A*}\left(t\right)$ and $E\left[T_{avg}\left(\pi^{*}\left(t\right)\right)\right]$ denote the corresponding workload and average TSD for fog node j, which can be achieved by the S-only policy [33]. Then, we have:

(37) $$\Delta L\left(t\right)+V\cdot E\left[T_{avg}\left(\pi \left(t\right)\right)\right]\leq \;B+E\left[\sum_{j=1}^{R}\left(Qw_{j}\left(t\right)\cdot \left(Xw_{j}^{A*}\left(t\right)-P_{j}\cdot \phi \right)\right)\right]+V\cdot E\left[T_{avg}\left(\pi^{*}\left(t\right)\right)\right].$$

We assume that the workload of the fog node is finite. Thus, there exists a finite constant $C=\max\left(E\left[Xw_{j}^{A*}\left(t\right)\right]\right)$. Let $\bar{T_{avg}^{*}}\left(t\right)=E\left[T_{avg}\left(\pi^{*}\left(t\right)\right)\right]$, we have:

(38) $$\Delta L\left(t\right)+V\cdot E\left[T\left(\pi \left(t\right)\right)\right]\leq B+\left(C-P_{j}\cdot \phi \right)\cdot E\left[\sum_{j=1}^{R}Qw_{j}\left(t\right)\right]+V\cdot \bar{T_{avg}^{*}}\left(t\right).\;$$

Summing both sides of the above inequality over T slots and disregarding the negative quantities, we have:

(39) $$E\left[L\left(T\right)\right]-E\left[L\left(0\right)\right]+V\cdot \sum_{t=0}^{T-1}E\left[T_{avg}\left(\pi^{*}\left(t\right)\right)\right]\;\leq T\cdot B+C\cdot \sum_{t=0}^{T-1}E\left[\sum_{j=1}^{R}Qw_{j}\left(t\right)\right]+V\cdot \sum_{t=0}^{T-1}\bar{T_{avg}^{*}}\left(t\right).\;$$

Owing to L(0)=0 and E[L(T)]≥0, we arrange the terms in the above inequality by dividing V·T as follows:

(40) $$\frac{1}{T}\cdot \sum_{t=0}^{T-1}E\left[T_{avg}\left(\pi \left(t\right)\right)\right]\leq \frac{B}{V}+\frac{1}{T}\cdot \sum_{t=0}^{T-1}\bar{T_{avg}^{*}}\left(t\right)+\frac{C}{T}\cdot \sum_{t=0}^{T-1}E\left[\sum_{j=1}^{R}Qw_{j}\left(t\right)\right].\;$$

Taking the limits as T→∞ and letting $\bar{T_{avg}^{*}}=\underset{T\to \infty }{\lim}\frac{1}{T}\cdot \sum_{t=0}^{T-1}\bar{T_{avg}^{*}}\left(t\right)$, we obtain the inequality as:

(41) $$\frac{1}{T}\cdot \sum_{t=0}^{T-1}E\left[T_{avg}\left(\pi \left(t\right)\right)\right]\leq \frac{B}{V}+\frac{1}{T}\cdot \sum_{t=0}^{T-1}\bar{T_{avg}^{*}}\left(t\right)+\frac{C}{T}\cdot \sum_{t=0}^{T-1}E\left[\sum_{j=1}^{R}Qw_{j}\left(t\right)\right].\;$$

Let $\bar{Qw^{max}}=\max\left(\underset{T\to \infty }{\lim}\frac{1}{T}\cdot \sum_{t=0}^{T-1}\sum_{j=1}^{R}E\left[Qw_{j}\left(t\right)\right]\right)$, then:

(42) $$\underset{T\to \infty }{\lim}\frac{1}{T}\cdot \sum_{t=0}^{T-1}E\left[T\left(\pi \left(t\right)\right)\right]\leq \frac{B}{V}+\bar{T_{avg}^{*}}+C\cdot \bar{Qw^{max}}.\;$$

Similarly, we assume that there exists a finite constant D such that $P_{j}\cdot \phi -Xw_{j}^{A*}\left(t\right)\leq D$ by the S-only policy [33]. Let $\bar{T_{avg}}\underset{T\to \infty }{=\lim\frac{1}{T}}\cdot \sum_{t=0}^{T-1}E\left[T_{avg}\left(\pi \left(t\right)\right)\right]$, in which case we obtain the following inequality by a similar process:

(43) $$\underset{T\to \infty }{\lim}\frac{1}{T}\cdot \sum_{t=0}^{T-1}E\left[\sum_{j=1}^{R}Qw_{j}\left(t\right)\right]\leq \frac{B+V\cdot \left[\bar{T_{avg}^{*}}-\bar{T_{avg}}\right]}{D}.\;$$

The bounds in Equations (42) and (43) indicate a $\left[O\left(\frac{1}{V}\right),O\left(V\right)\right]$ tradeoff between the average TSD and average queuing length of a workload. The average TSD approaches the DAOWA-generated $\bar{T_{avg}^{*}}$ when parameter V is sufficiently large, but ignores the stability of the average queue of a workload in the fog node. By contrast, the average queuing length of a workload approaches its optimum when the value of V is small. Tuning the parameter V can achieve the optimal objective of minimizing $\bar{T_{avg}^{*}}$ as well as guaranteeing the stability of queuing the workload of the fog nodes. 

## 6. Performance Evaluation

### 6.1. Simulation Environment Settings

We choose CloudSim [34] as our simulation platform, and we extended CloudsSim by adding new settings to conduct our experiments, which are similar to those in previous reports [35,36]. The simulation scenario comprises three regions and a cloud center, with each region endowed with one fog node and a number of IoT end devices. Simulation parameters and the topology are listed in Table 1. Based on [37], we also use the Poisson distribution with the vector $\lambda \left(t\right)=\left[\bar{\lambda_{1}\left(t\right)},\;\bar{\lambda_{2}\left(t\right)},\;\bar{\lambda_{3}\left(t\right)}\right]$ representing the expected number of arrivals to model the task generation rate of the end tier in the three regions. For each time slot, the length of the corresponding task instruction (million instructions, MI) follows an exponential distribution with an expected length vector $l\left(t\right)=\left[\bar{l_{1}\left(t\right)},\;\bar{l_{2}\left(t\right)},\;\bar{l_{3}\left(t\right)}\right]$. Similarly, the data size follows a uniform distribution with an average size vector $d\left(t\right)=\left[\bar{d_{1}\left(t\right)},\;\bar{d_{2}\left(t\right)},\;\bar{d_{3}\left(t\right)}\right]$. We set the frequency of each central processing unit (CPU) cycle of VMs in the cloud center to 3.2 GHz, which is faster than that of each fog node (2.0 GHz). The *CPI* of both the cloud center and the fog node are 2.5. Based on the real situation, the f2f bandwidth is 54 Mbps, whereas the f2c bandwidth is 1 Gbps. The mean f2f and f2c propagation delays are 1 ms and 50 ms, respectively.

### 6.2. Impact of V on Average Task Service Delay

**Figure 3:**
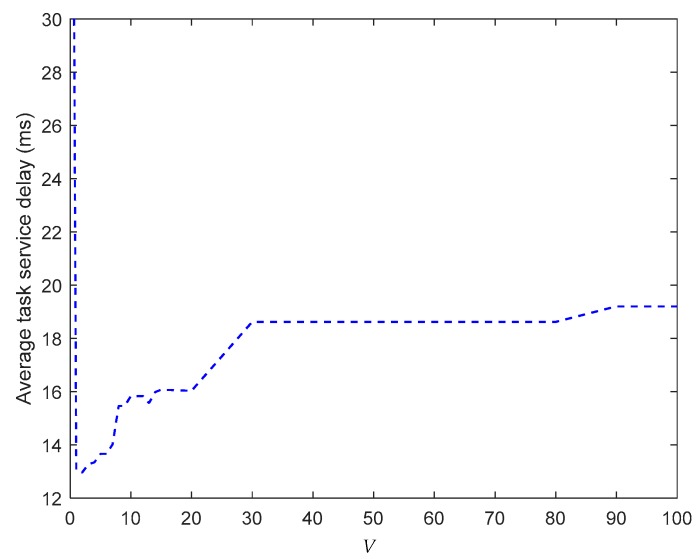
Average task service delay vs. *V.*

Figure 3 shows the impact of control parameter V, which is defined in Equation (29), on the average TSD. When V=0, the proposed algorithm degrades into a workload-based Lyapunov workload allocation approach. According to the theoretical analysis, the policy should stabilize $Qw_{j}\left(t\right)$, and the best scheme should ensure that $Qw_{j}\left(t\right)=0$. Thus, the optimal policy schedules all tasks for processing in the cloud center. However, owing to the large f2c propagation delay, it is unsurprising that the average TSD is the largest in comparison with those obtained when V>0. When 0<V<1, the average TSD first decreases as the value of V increases. When 1<V<10, the average TSD reaches a small value. This is because our proposed algorithm attempts to find the optimal workload allocation and task scheduling policy based on the tradeoff of the penalty over the workload in the drift-plus-penalty formulation. The average TSD starts to increase when V>10, because the larger V is, the lower the $Qw_{j}\left(t\right)$ affection is. Thus, it increases the queuing delay as $Qw_{j}\left(t\right)$ increases. The result also verifies the analysis in Equation (42), where longer $\bar{Qw^{max}}$ leads to a larger bound for the average TSD.

### 6.3. Comparison of the Task Service Delay of the Regions

**Figure 4:**
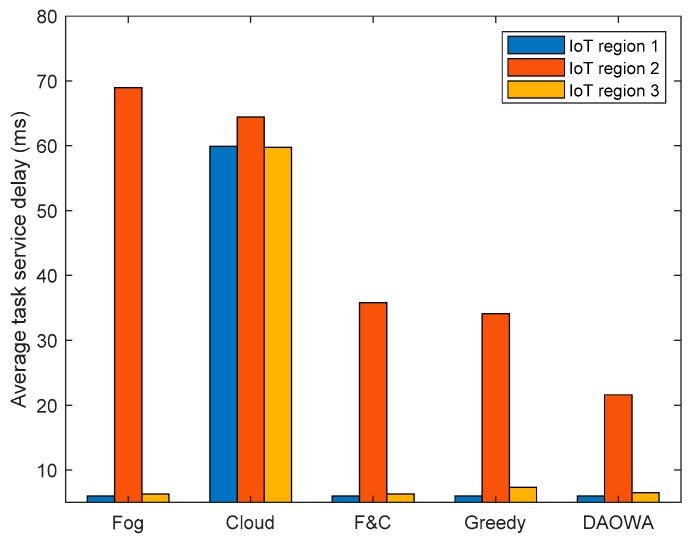
Average task service delay for each of the three IoT regions computed with the different algorithms.

We evaluated the efficiency of the proposed DAOWA algorithm by comparing with other algorithms, including fog-processing algorithm (Fog), cloud processing algorithm (Cloud), fog-to-cloud cooperation algorithm (F&C) and greedy algorithm (Greedy). In the Fog algorithm, all tasks are processed at the local fog node. In the Cloud algorithm, all tasks are offloaded to the cloud center. The F&C algorithm decides whether the task should be processed at the local fog node or offloaded to the cloud center with the aim of minimizing the delay. The Greedy algorithm only considers the TSD at the current time slot as the objective to determine the workload allocation decision. The average TSD of the three regions defined in Table 1 was computed by the aforementioned four algorithms and is plotted in Figure 4.

With reference to Table 1, because the task arrival rate and task instruction length in IoT region 2 are larger than those in the other two IoT regions, the large workload arrival at IoT region 2 yields the largest average TSD. Because the tasks can only be processed at the local fog node when using the Fog algorithm, the limited computation capability of the fog node and the large workload arrival at IoT region 2 causes significant queuing delay to drastically increase the average TSD. Although the cloud center has sufficient computation capability to eliminate the queuing delay, the large f2c propagation delay increases the average TSD of each IoT region. Therefore, the results in Figure 4 demonstrate that it is vital to enable the fog node to cooperate with the cloud. The advantage is also demonstrated by the result of the F&C algorithm. Compared with the F&C algorithm, although the Greedy algorithm achieves a lower TSD, it ignores the workload stability of the fog node, which may cause a long queuing delay to limit the average TSD reduction. Our proposed algorithm (i.e., DAOWA) can improve the average TSD compared with the F&C algorithm. This is because the DAOWA algorithm not only enables the local fog node to cooperate with the cloud center, it also enables the local fog node to cooperate with its neighboring fog nodes. Furthermore, the tradeoff between the queuing delay and network delay is optimized by the DAOWA algorithm. Thus, the average TSD can be reduced significantly by the DAOWA algorithm.

### 6.4. Varying the Task Arrival Rate

**Figure 5:**
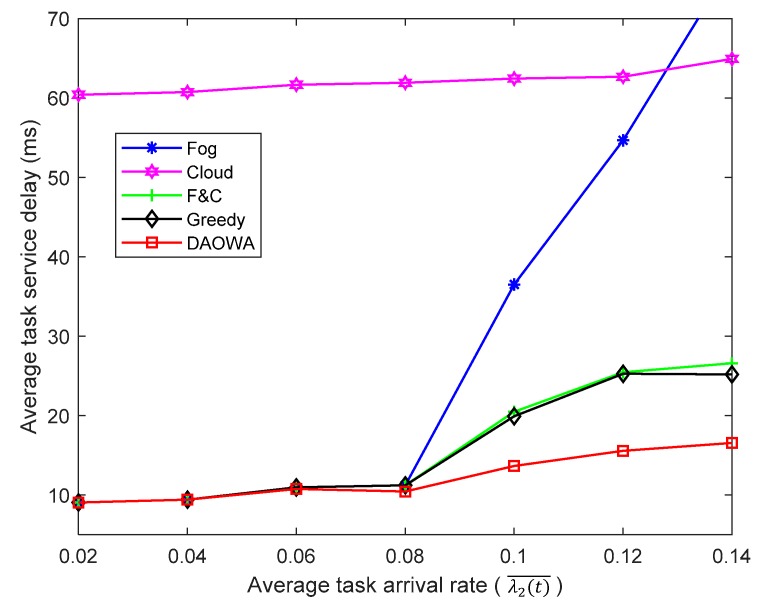
Average task service delay vs. task arrival rate.

We evaluated the efficiency of the proposed algorithm for different task arrival rates. Based on Table 1, we set $\bar{\lambda_{1}\left(t\right)}=\bar{\lambda_{3}\left(t\right)}=0.5\bar{\lambda_{2}\left(t\right)}$.The value of V was set to 5. Then, we vary the task arrival rate of IoT region 2 from 0.02 to 0.14. The results are shown in Figure 5.

When the task arrival rate is low ($\bar{\lambda_{2}\left(t\right)}<0.08$), the computation capability of the local fog node is sufficient to guarantee the stability of $Qw_{j}\left(t\right)$. In this situation, the optimal policy enables the tasks to be processed at the local fog node rather than offloading them to other fog nodes or the cloud center. Thus, the F&C and Greedy algorithms allocate most of the workload to the local fog node, depending on the delay between the local fog node and the cloud center. The DAOWA algorithm also allocates most of the workload to the local fog node. Thus, all the algorithms achieve similar TSDs.

When the task arrival rate increases ($\bar{\lambda_{2}\left(t\right)}>0.08$)), the Fog algorithm causes severe queuing delays owing to the limited computation capability of the fog node. Thus, the Fog algorithm exacerbates the average TSD. Furthermore, when the task arrival rate is sufficiently large (e.g.,$\;\bar{\lambda_{2}\left(t\right)}>0.12$), the average TSD obtained by the Fog algorithm could be larger than that obtained by the Cloud algorithm. Because the F&C algorithm enables the local fog node to cooperate with the cloud center, it succeeds in lowering the average TSD. However, the f2c propagation delay affects the performance of the F&C algorithm. The average TSD obtained with the F&C algorithm increases when the task arrival rate increases. The Greedy algorithm ignores the workload stability of the fog node, and a long queuing delay may impair the advantage of the fog-to-fog coordination. Thus, the performance of the Greedy algorithm is similar to that of the F&C algorithm. Compared with the other algorithms, the DAOWA algorithm is not only aware of the workload of the fog node, but also considers the network delay. It guarantees the workload stability of the fog node and avoids a large network delay. This means that the DAOWA algorithm is able to adaptively achieve the lowest average TSD according to the task arrival rate. In spite of this, the increasing task arrival rate results in more tasks being offloaded to the cloud center. In this sense, the large f2c propagation delay increases the average TSD.

Because of the large propagation delay, the average TSD obtained by the Cloud algorithm is larger than that obtained by the F&C, Greedy and DAOWA algorithms. Because of the large f2c bandwidth and the sufficient computation capability of the cloud center, the average TSD obtained with the Cloud algorithm changes slowly when using a one user-one VM mode.

### 6.5. Varying the Task Instruction Length

**Figure 6:**
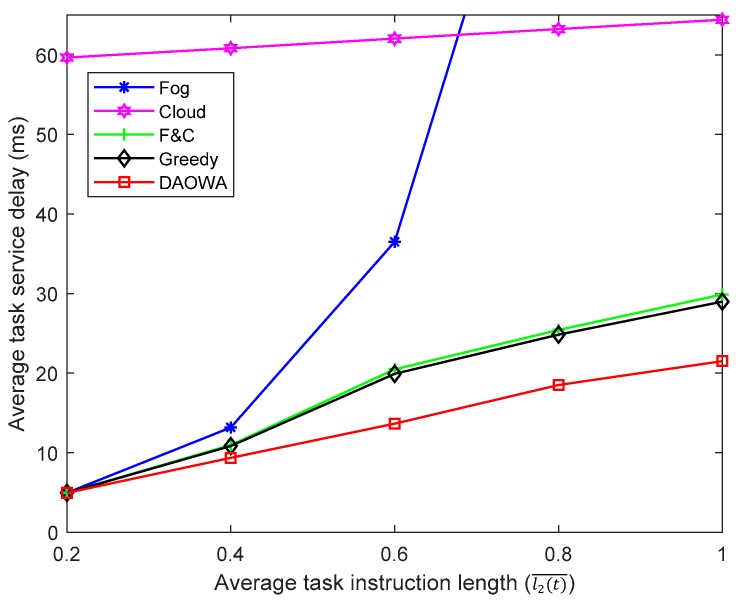
Average task service delay vs. task instruction length

We also evaluate the efficiency of the Fog, Cloud, F&C, Greedy and DAOWA algorithms for various task instruction lengths. We adopt the parameter settings listed in Table 1 except for the average task instruction length. We set $\bar{l_{1}\left(t\right)}=\bar{l_{3}\left(t\right)}=0.5\bar{l_{2}\left(t\right)}$, that is, the expected task length in IoT region 1 and IoT region 3 is half of that in IoT region 2. The value of V is set to 5. Then, we vary the expected task length of IoT region 2 from 0.2×10 MI to 1×10 MI. The results are shown in Figure 6.

When the task instruction length is short ($\bar{l_{2}\left(t\right)}<0.4$), the computation capability of the local fog node can guarantee the workload stability for the low delay requirement. In this situation, most of the workloads do not need to be offloaded to the neighboring fog node and the cloud center. Thus, the performance of the Fog, F&C, Greedy and DAOWA algorithms is similar. However, owing to the large f2c propagation delay, the Cloud algorithm obtains the largest average TSD.

When the average task instruction increases ($\bar{l_{2}\left(t\right)}>0.4$), the limited computation capability of the fog node is unable to stabilize $Qw_{j}$, whereupon the queuing delay increases markedly. Furthermore, a significant increase in the average task instruction length causes the queuing delay to exceed the f2c propagation delay, in which case the average TSD of the Fog algorithm becomes larger than that of the Cloud algorithm. Without fog-to-fog cooperation, the F&C algorithm offloads additional work to the cloud center, which increases the propagation and congestion delay, leading to a larger average TSD than that of the DAOWA algorithm. Similar with the experiment in Section 6.4, a long queuing delay under the Greedy algorithm may limit the advantage of the fog-to-fog coordination. Thus, the average TSD under the Greedy algorithm is similar to that under the F&C algorithm. Overall, regardless of the workload, the DAOWA algorithm always provides the lowest average TSD in comparison with other algorithms, as shown in Figure 6. This is because the DAOWA algorithm can dynamically switch among the local fog node, neighboring fog nodes, and the cloud center adaptively to guarantee the stability of the workload queue and minimize the TSD for various task instruction lengths.

### 6.6. Varying the Computing Speed of the Fog Node

**Figure 7:**
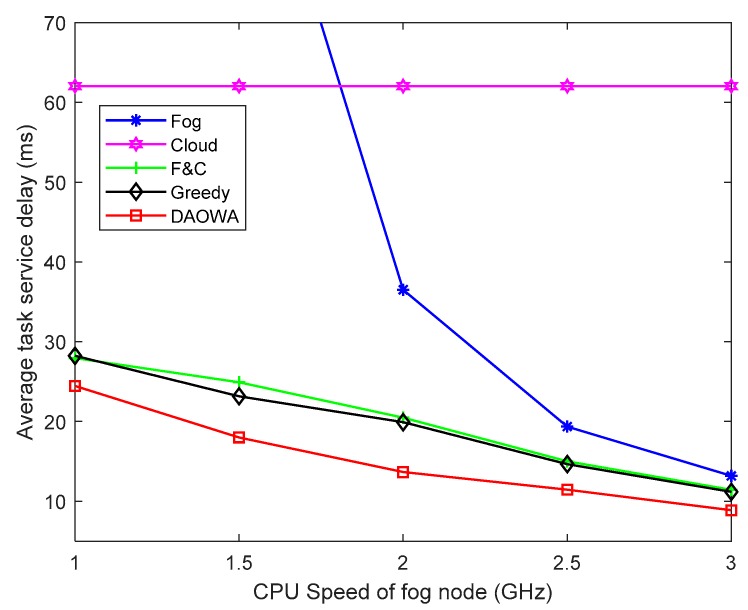
Average task service delay vs. fog node computing speed.

To evaluate the efficiency of our proposed algorithm, we vary the frequency of the CPU cycle of the fog nodes from 1.0 GHz to 3.0 GHz according to the definition of computing speed in Equation (1). The other parameter settings are the same as those in Table 1. The results of this comparison are plotted in Figure 7.

Unsurprisingly, when the computing speed of the fog node is low ($F_{j}^{fog}<2.0\;\mathrm{GHz}$), the poor computation capability of the fog node causes significant queuing delay. Consequently, the average TSD obtained by the Fog algorithm is enormous, even larger than the average TSD of the Cloud algorithm. The F&C algorithm enables the local fog node to cooperate with the cloud center to mitigate the poor performance of the local fog node. The DAOWA algorithm achieves the lowest average TSD by the help of neighboring fogs. Although the Greedy algorithm also enables the local fog node to cooperate with its neighboring fog nodes and the cloud center, it ignores the workload stability. Furthermore, because the heavy workload of the fog nodes under the Greedy algorithm will cause a long queuing delay, the average TSD under the Greedy algorithm is larger than that under the DAOWA algorithm.

Increasing the computing speed of the fog node ($F_{j}^{fog}>2.0\;\mathrm{GHz}$) leads to an increase in its performance, which reduces the average TSD when using the Fog, F&C, and DAOWA algorithms. Compared with other algorithms, the DAOWA algorithm can adaptively allocate the workload according to the computing speed of the fog node. The DAOWA algorithm stabilizes the workload and avoids large processing delays. Thus, the DAOWA algorithm obtains the lowest average TSD. Furthermore, when $F_{j}^{fog}=3.0\;\mathrm{GHz}$, because of the considerable increase in the computing speed of the fog node, the DAOWA algorithm does not need to enable the local fog node to cooperate with other fog nodes. Thus, the result of the DAOWA algorithm is similar to that of the Fog, F&C and Greedy algorithms. In real situations, it would not be possible to significantly increase the computing speed of the fog node.

### 6.7. Varying the f2c Propagation Delay

**Figure 8:**
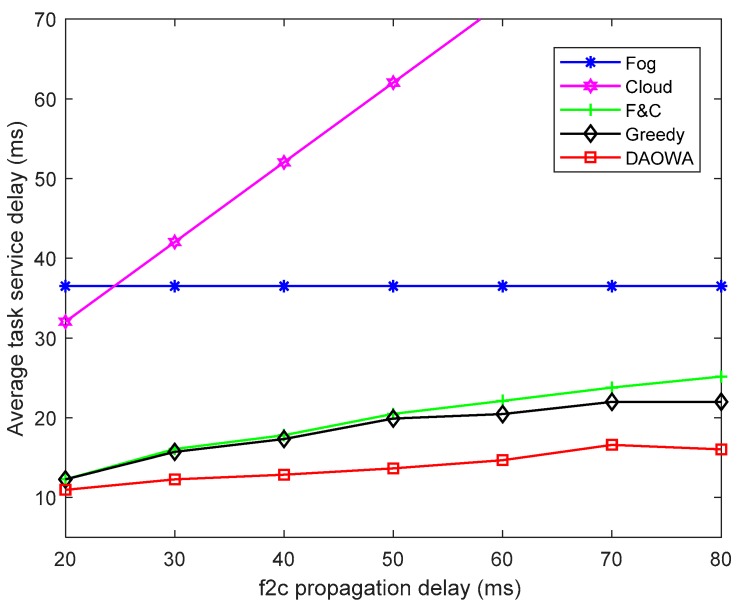
Average task service delay vs. f2c propagation delay.

We evaluated the efficiency of our proposed algorithm by varying the f2c propagation delay from 20 ms to 80 ms. The other parameter settings are the same as those in Table 1. The results are shown in Figure 8.

As shown in Figure 8, because the Fog algorithm cannot be affected by the f2c propagation delay, the average TSD of the Fog algorithm does not change. Furthermore, owing to the limited computation capability of the fog node, the average TSD calculated with the Fog algorithm is larger than that obtained by the F&C, Greedy and DAOWA algorithms. In addition, the average TSD of the Cloud algorithm increases when the f2c propagation delay increases. This demonstrates that the f2c propagation delay mainly affects the average TSD of the Cloud algorithm because of the long-distance data transmission between the fog node and cloud center. However, owing to the cooperation between the local fog node and cloud center, the average TSD of the F&C algorithm is less than that obtained by the Cloud and Fog algorithms.

When the f2c propagation delay is small (20 ms), the average TSD under the F&C and Greedy algorithms are lower than that of the Fog algorithm, and are close to that of the DAOWA algorithm. This is because the computation capability of the cloud center is sufficient, the DAOWA and Greedy algorithms do not need to allocate the workload to neighboring fog nodes when the f2c propagation delay is small. In this case the optimized scheme enables the local fog node to cooperate directly with the cloud center. Another noteworthy observation is that the average TSD of the F&C, Greedy and DAOWA algorithms increases as the f2c propagation delay increases. When the f2c propagation delay increases, the Greedy algorithm will enable the local fog node to cooperate with its neighboring fog nodes to prevent the average TSD from increasing fast. Thus, the average TSD under the Greedy algorithm is lower than that under the F&C algorithm. The average TSD under the DAOWA algorithm is the lowest. This demonstrates the ability of the DAOWA algorithm to optimize the policy to allocate the workload according to the system status.

## 7. Conclusions

This paper proposes a fog-to-fog cooperation scheme to minimize the task-processing delay in an IoT-fog-cloud system. Based on the topology framework of the system in real situations, we have built a systematic, comprehensive, and analytic time-varying queuing model. This model considers the computation capability, the amount of traffic, and the network transmission delay. In particular, we have formulated and developed a delay-aware workload allocation scheme, named DAOWA. We have analyzed the Lyapunov drift-plus-penalty properties of the time-varying queuing model of the fog tier and design the algorithm to minimize the drift-plus-penalty to reduce the average TSD. The theoretical analysis and simulation results demonstrate the ability of the proposed algorithm can minimize the task service delay efficiently.

## Figures and Tables

**Table 1:** The basic parameter settings.

Parameters	IoT Region 1	IoT Region 2	IoT Region 3
$F_{j}^{fog}$(*GHz*)	2.0	2.0	2.0
*CPI*	2.5	2.5	2.5
CPUs	1	1	1
$\bar{\lambda_{j}\left(t\right)}$	$0.5\bar{\lambda_{2}\left(t\right)}$	0.10	$0.5\bar{\lambda_{2}\left(t\right)}$
$\bar{l_{j}}\left(t\right)$(10MI)	$0.5\bar{l_{2}\left(t\right)}$	0.6	$0.5\bar{l_{2}\left(t\right)}$
$\bar{d_{j}\left(t\right)}$(Mbits)	U[1, 10]	U[1, 10]	U[1, 10]
Neighbor	Region 2	Region 1, 3	Region 2
$F^{cloud}$(GHz)	3.2		
*CPI*	2.5		
f2f bandwidth (Mbps)	54		
f2c bandwidth (Gbps)	1		
f2f propagation delay (ms)	1		
f2c propagation delay (ms)	50		

## Appendix A

**Proof** **of** **Theorem** **1.** By squaring both sides of Equation (5), we can obtain:

$${\left[Qw_{j}\left(t+1\right)\right]}^{2}={\left\{\max\left[Qw_{j}\left(t\right)+Xw_{j}^{A}\left(t\right)-P_{j}\cdot \phi ,0\right]\right\}}^{2}.\;$$

Then,

$${\left[Qw_{j}\left(t+1\right)\right]}^{2}\leq {\left[Qw_{j}\left(t\right)+Xw_{j}^{A}\left(t\right)-P_{j}\cdot \phi \right]}^{2}$$

We have

$${\left[Qw_{j}\left(t+1\right)\right]}^{2}\leq \left\{{\left[Qw_{j}\left(t\right)\right]}^{2}+2\cdot Qw_{j}\left(t\right)\cdot \left[Xw_{j}^{A}\left(t\right)-P_{j}\cdot \phi \right]+{\left[Xw_{j}^{A}\left(t\right)-P_{j}\cdot \phi \right]}^{2}\right\}.\;$$

According to the definition of Lyapunov drift in Equation (27), we have the following:

$$\Delta L\left(t\right)=E\left[\frac{1}{2}\sum_{j=1}^{R}{\left[Qw_{j}\left(t+1\right)\right]}^{2}-\frac{1}{2}\sum_{j=1}^{R}{\left[Qw_{j}\left(t\right)\right]}^{2}|Qw\left(t\right)\right]\;\leq \frac{1}{2}E\left[\sum_{j=1}^{R}{\left(Xw_{j}^{A}\left(t\right)-P_{j}\cdot \phi \right)}^{2}|Qw\left(t\right)\right]+E\left[\sum_{j=1}^{R}\left(Qw_{j}\left(t\right)\cdot \left(Xw_{j}^{A}\left(t\right)-P_{j}\cdot \phi \right)\right)|Qw\left(t\right)\right].$$

Because the number of tasks and the length of task instructions in the fog system are finite, $Xw_{j}^{A}\left(t\right)$ has its upper bound $Xw_{j}{}^{max}\left(t\right)$, i.e., $Xw_{j}^{A}\left(t\right)\leq Xw_{j}{}^{max}\left(t\right)$. Furthermore, the computing speed of the fog node can be considered to be a constant value. Thus,

$${\left[Xw_{j}^{A}\left(t\right)-P_{j}\cdot \phi \right]}^{2}\leq max\left\{{\left[Xw_{j}{}^{max}\left(t\right)\right]}^{2},\;{\left[P_{j}\cdot \phi -Xw_{j}^{A}\left(t\right)\right]}^{2}\right\}.$$

Let $B=\max\left\{\frac{1}{2}{\left[Xw_{j}{}^{max}\left(t\right)\right]}^{2},\;\frac{1}{2}{\left[P_{j}\cdot \phi -Xw_{j}^{A}\left(t\right)\right]}^{2}\right\}$. Subsequently, we have

$$\Delta L\left(t\right)\leq B+E\left[\sum_{j=1}^{R}\left(Qw_{j}\left(t\right)\cdot \left(Xw_{j}^{A}\left(t\right)-P_{j}\cdot \phi \right)\right)|Qw\left(t\right)\right],\;$$

which proves Theorem 1. □
